# Supplementary material for: Recovery of consciousness and cognition after general anesthesia in humans
Source: eLife. 2021 May 10;10:e59525. doi: 10.7554/eLife.59525 (PMC8163502; doi:10.7554/eLife.59525)
Supplement: Supplementary file 1. [file elife-59525-supp1.docx]

**Supplementary file 1A.** Sample size of data epochs in EEG analysis

| **Epochs** | **Anesthetized Group** | **Control Group** |
| --- | --- | --- |
| EC1 | n=29 | n=28 |
| LOC | n=30 | -- |
| Maintenance | n=29 | -- |
| Pre-ROC | n=28 | -- |
| EC2 | n=28 | n=30 |
| EC3 | n=28 | n=30 |
| EC4 | n=29 | n=29 |
| EC5 | n=29 | n=30 |
| EC6 | n=30 | n=30 |
| EC7 | n=29 | n=30 |

**Supplementary file 1B.** Model selection for the statistical analysis of EEG measures

| **EEG measures** | **LMM models** | **Number of parameters** | **AIC** | **BIC** | **-2 Restricted Log Likelihood** | **LST statistics** |
| --- | --- | --- | --- | --- | --- | --- |
| **PE** | The employed model | 31 | -2143.831 | -2096.263 | -2165.831 | -- |
|  | The model with random center effect | 33 | -2145.203 | -2088.986 | -2171.203 | $\chi^{2}\left( 2 \right)$=5.372, p=0.068 |
|  | The model with region as additional repeated effect | 41 | -2133.480 | -2042.669 | -2175.480 | $\chi^{2}\left( 10 \right)$=9.649, p=0.472 |
|  | The model with AR1 as the covariance structure of the residuals | 23 | -2073.166 | -2060.193 | -2079.166 | $\chi^{2}\left( 8 \right)$=86.665, p<0.001 |
| **LZC** | The employed model | 21 | -779.126 | -739.182 | -801.126 | -- |
|  | The model with random center effect | 23 | -779.732 | -732.526 | -805.732 | $\chi^{2}\left( 2 \right)$=4.606, p=0.100 |
|  | The model with AR1 as the covariance structure of the residuals | 13 | -714.628 | -703.734 | -720.628 | $\chi^{2}\left( 8 \right)$=80.498, p<0.001 |
