# Supplementary material for: Recovery of consciousness and cognition after general anesthesia in humans
Source: eLife. 2021 May 10;10:e59525. doi: 10.7554/eLife.59525 (PMC8163502; doi:10.7554/eLife.59525)
Supplement: Transparent reporting form [file elife-59525-transrepform.docx]

***eLife’s* transparent reporting form**

We encourage authors to provide detailed information *within their submission* to facilitate the interpretation and replication of experiments. Authors can upload supporting documentation to indicate the use of appropriate reporting guidelines for health-related research (see [EQUATOR Network](about:blank)), life science research (see the BioSharing Information Resource), or the [ARRIVE guidelines](about:blank) for reporting work involving animal research. Where applicable, authors should refer to any relevant reporting standards documents in this form.

If you have any questions, please consult our Journal Policies and/or contact us: [editorial@elifesciences.org](about:blank).

**Sample-size estimation**

- You should state whether an appropriate sample size was computed when the study was being designed
- You should state the statistical method of sample size computation and any required assumptions
- If no explicit power analysis was used, you should describe how you decided what sample (replicate) size (number) to use

Please outline where this information can be found within the submission (e.g., sections or figure legends), or explain why this information doesn’t apply to your submission:

Information on the choice of appropriate sample size is found on page 18 of the manuscript and is copied here for convenience. “The main factor that was considered in estimating our required sample size was the time difference in return of cognitive functions within the subjects receiving general anesthesia. Sample size calculation was modeled with various assumptions regarding the difference in recovery times between the first and last cognitive domains to return, and the standard deviations of these parameters. A range between 30 min and 90 min was considered for differences in recovery times between cognitive domains (possible effect sizes). A range between 20 min and 40 min was considered for standard deviations of these parameters. Assuming relatively conservative estimates (difference in recovery times = 30 min and standard deviation = 40 min), 30 subjects would provide >80% power with a two-sided alpha<0.05, using an unpaired t test. With relatively liberal assumptions (difference in recovery times = 90 min and standard deviation = 20 min), 30 subjects would provide >99% power with a two-sided alpha<0.001, using an unpaired t test.”

**Replicates**

- You should report how often each experiment was performed
- You should include a definition of biological versus technical replication
- The data obtained should be provided and sufficient information should be provided to indicate the number of independent biological and/or technical replicates
- If you encountered any outliers, you should describe how these were handled
- Criteria for exclusion/inclusion of data should be clearly stated
- High-throughput sequence data should be uploaded before submission, with a private link for reviewers provided (these are available from both GEO and ArrayExpress)

Please outline where this information can be found within the submission (e.g., sections or figure legends), or explain why this information doesn’t apply to your submission:

All information about the replicates is found in the methods section. Specific information on the inclusion and exclusion criteria for this study has been previously published in Maier et al., Frontiers in Human Neuroscience 2017 Volume 11, article 284 and is referenced in the bibliography section of this submission. Information on the number of biological replicates (n=30 anesthetized and n=30 awake controls) is found in the methods section under participants. In the instance of an “outlier,” for example in Figure 4, data are nevertheless reported and denoted with a red cross.

**Statistical reporting**

- Statistical analysis methods should be described and justified
- Raw data should be presented in figures whenever informative to do so (typically when N per group is less than 10)
- For each experiment, you should identify the statistical tests used, exact values of N, definitions of center, methods of multiple test correction, and dispersion and precision measures (e.g., mean, median, SD, SEM, confidence intervals; and, for the major substantive results, a measure of effect size (e.g., Pearson's r, Cohen's d)
- Report exact p-values wherever possible alongside the summary statistics and 95% confidence intervals. These should be reported for all key questions and not only when the p-value is less than 0.05.

Please outline where this information can be found within the submission (e.g., sections or figure legends), or explain why this information doesn’t apply to your submission:

Information on statistics is found in the methods section.

(For large datasets, or papers with a very large number of statistical tests, you may upload a single table file with tests, Ns, etc., with reference to sections in the manuscript.)

**Group allocation**

- Indicate how samples were allocated into experimental groups (in the case of clinical studies, please specify allocation to treatment method); if randomization was used, please also state if restricted randomization was applied
- Indicate if masking was used during group allocation, data collection and/or data analysis

Please outline where this information can be found within the submission (e.g., sections or figure legends), or explain why this information doesn’t apply to your submission:

Information on group allocation is found in the methods section under “participants.”

**Additional data files (“source data”)**

- We encourage you to upload relevant additional data files, such as numerical data that are represented as a graph in a figure, or as a summary table
- Where provided, these should be in the most useful format, and they can be uploaded as “Source data” files linked to a main figure or table
- Include model definition files including the full list of parameters used
- Include code used for data analysis (e.g., R, MatLab)
- Avoid stating that data files are “available upon request”

Please indicate the figures or tables for which source data files have been provided:

Instructions on Matlab code and actual scripts used are all freely available in the signal processing toolbox as described in the methods section under EEG analysis or in the permutation entropy and Lempel-Ziv Complexity sections of the Methods. The fit model for cognitive data are fully presented in the statistical analysis of cognitive data section of the methods.
